# Atypical acute *Helicobacter pylori* infection without atrophic gastritis: A case report

**DOI:** 10.20407/fmj.2025-050

**Published:** 2026-05-14

**Authors:** Ryuzo Deguchi, Haruhiko Ogata

**Affiliations:** Department of Gastroenterology, Fujita Health University Haneda Clinic, Ota, Tokyo, Japan

**Keywords:** Primary *Helicobacter pylori* infection, Urea breath test, Anti-*H. pylori* antibodies, Smart Gene, Atrophic gastritis

## Abstract

A 64-year-old Chinese man visited our clinic’s health-screening center. The patient tested negative for *Helicobacter pylori* (*HP*) infection in his home country, but at this visit, anti-*HP* antibodies of 5 U/mL and a urea breath test (UBT) result of 48.6‰ were detected. Upper gastrointestinal endoscopy showed no atrophic gastritis, and a regular arrangement of collecting venules was observed. Since a false-positive UBT was also considered, Smart Gene testing was performed, which confirmed the *HP* positivity. As no clarithromycin resistance mutation was detected, Japan’s first-line eradication regimen was prescribed. The eradication test result was a UBT of 0.1‰, confirming successful *HP* eradication.

## Introduction

Chronic persistent infection with *Helicobacter pylori* (*HP*) is thought to cause a stepwise progression from atrophic gastritis to intestinal metaplasia to dysplasia to adenocarcinoma.^1^ A meta-analysis of the global prevalence of *HP* infection reported a worldwide rate of 44.3%,^2^ but significant variations in frequency exist among countries.^3^ East Asia is noted for its high incidence of gastric cancer, with China reported to have the highest incidence, prevalence, and mortality rates among these countries.^4^ Chronic persistent infection with *HP* creates a background gastric mucosa that is prone to gastric cancer development, but reports from Japan indicate that eradicating *HP* reduces gastric cancer incidence.^5,6^

Generally, *HP* transmission is thought to occur through childhood oral infection and intra-family transmission,^7^ and reinfection is rare.^8^ Serological testing is one method for diagnosing *HP* infection, but some countries do not recommend it in their guidelines because it cannot distinguish between active infection and past infection.^9,10^ However, some countries do recommend it under certain conditions with various restrictions, considering cost-effectiveness.^11,12^ A report analyzing guidelines on *HP* worldwide indicated that urea breath testing is widely recommended as the first-line diagnostic method for *HP* infection.^13^ A case of initial active *HP* infection in an adult with atypical endoscopic findings, detected by the urea breath test (UBT), is reported.

## Case report

**Figure 1:**
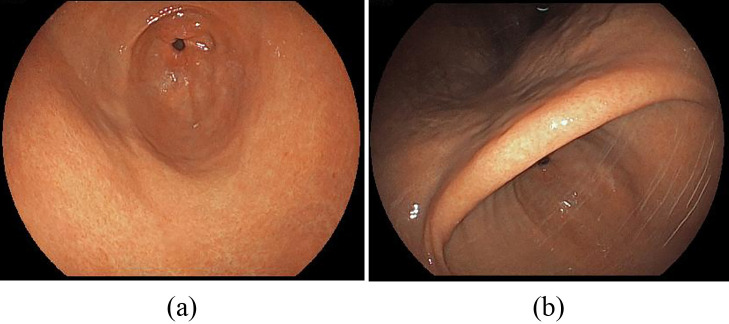
(a) No atrophic changes are observed in the gastric antrum. (b) A distinct regular arrangement of collecting venules (RAC) is observed in the lesser curvature of the gastric angle.

**Figure 2:**
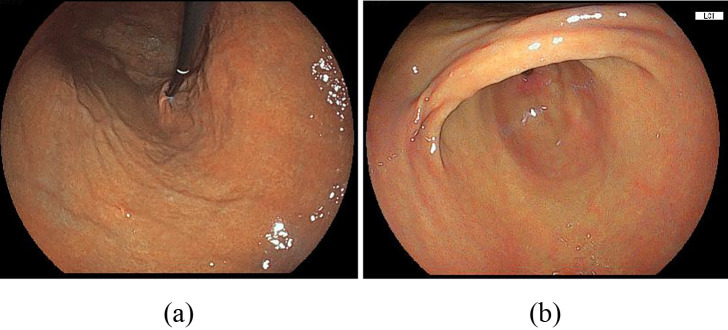
(a) Mild red streaks are observed in the lesser curvature of the gastric body, but no atrophic changes are present. (b) Mild red streaks are observed in the greater curvature of the gastric antrum (Linked Color Imaging (LCI) mode).

**Figure 3:**
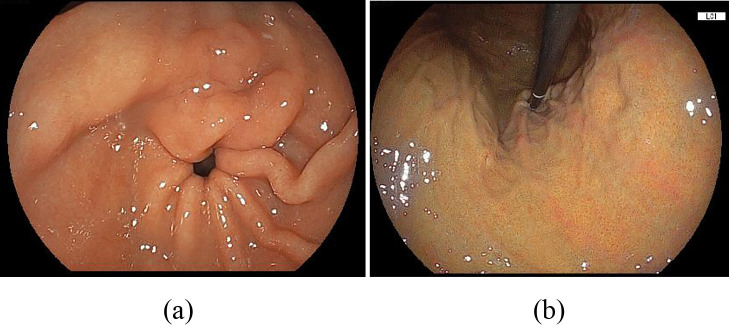
(a) No obvious abnormalities are observed around the pyloric ring. (b) In Linked Color Imaging (LCI) mode, the background mucosa of the body exhibits an apricot-colored hue.

**Figure 4:**
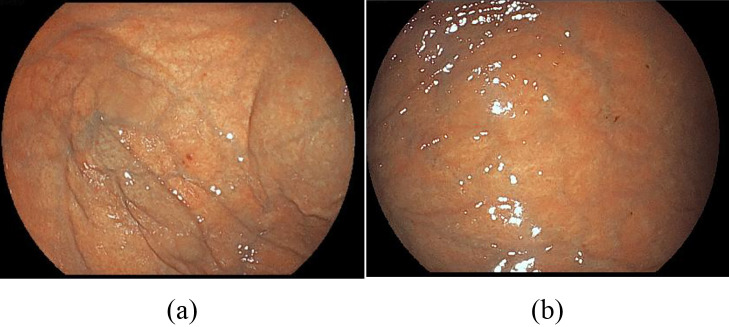
(a) A slight spotty redness is observed in the gastric fornix. (b) A slight spotty redness is observed in the greater curvature of the gastric body.

**Figure 5:**
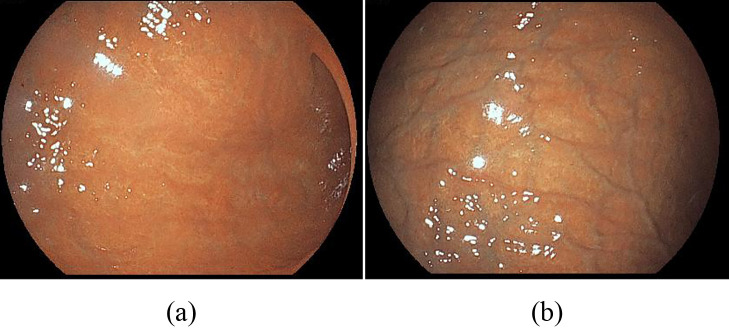
(a) No enlarged folds are observed in the lower greater curvature of the gastric body. (b) No enlarged folds or mucosal swelling are observed in the upper greater curvature of the gastric body.

A 64-year-old Chinese man (Linyi City, Shandong Province) visited our clinic’s health-screening center. He underwent an upper gastrointestinal endoscopy (UGE) examination during a health checkup at another hospital in 2023, which revealed no abnormalities, and *HP* infection was negative based on serum antibody testing. However, at the visit to our clinic, anti-*HP* antibodies (H. pylori-latex Seiken, Denka Seiken Co., Ltd., Tokyo, Japan) of 5 U/mL (negative <10 U/mL) and a positive UBT of 48.6‰ (positive ≥2.5‰) were detected. His spouse (a 59-year-old Chinese woman), who underwent examination simultaneously, had a history of *HP* eradication therapy. Her anti-*HP* antibody titer was 7 U/mL, and her UBT result was 0.2‰. UGE showed atrophic changes in the antrum, with a sparse regular arrangement of collecting venules (RAC). UBT positivity with anti-*HP* antibodies was judged as negative-high titer, so UGE was performed. No atrophic changes were observed in the gastric antrum (Figure 1a), and RAC was detected (Figure 1b). A mild red streak was observed in the gastric body (Figure 2a) and antrum (Figure 2b). No obvious abnormalities were observed around the pyloric ring (Figure 3a), and the background mucosa of the body appeared apricot-colored in Linked Color Imaging mode (Figure 3b). A slight spotty redness was observed in the gastric fornix and greater curvature of the body (Figure 4a, b). No enlarged folds were observed in the lower greater curvature of the gastric body (Figure 5a), nor were any enlarged folds or mucosal swelling observed in the upper greater curvature of the gastric body (Figure 5b). No findings suggestive of active *HP* infection were observed on UGE, and although a repeat UBT was considered, gastric fluid was collected and tested using Smart Gene (MIZUHO MEDY Co., Ltd., Tosu-City, Saga, Japan). *HP*-positive status and no clarithromycin resistance were confirmed, so Japan’s first-line *HP* eradication therapy (20 mg of vonoprazan, 750 mg of amoxicillin, and 200 mg of clarithromycin twice daily for 7 days) was prescribed. Five weeks after completing oral eradication therapy, a UBT result of 0.1‰ and anti-*HP* antibody concentration of 5 U/mL were confirmed. *HP* primary eradication was considered successful, and follow-up plans were made for the next year’s health check to include UGE, anti-*HP* antibody testing, and UBT.

## Discussion

This case did not suggest active *HP* infection based on the anti-*HP* antibody and UGE results. This case was diagnosed as active *HP* infection without considering that the UBT result could be a false-positive result, since both the UBT and Smart Gene tests were positive. Regarding the route of *HP* infection in this patient, the absence of atrophic changes in the gastric mucosa during UGE and the presence of RAC suggested that infection during childhood was unlikely. Regarding his spouse, considering her history of *HP* eradication, negative anti-*HP* antibodies, and negative UBT, she was considered to have had a pre-existing infection followed by successful *HP* eradication. Although horizontal transmission from a spouse is unlikely, the absence of UGE findings and elevated antibody titers in this patient suggest that a recent, unexplained infection was the most probable scenario.

Regarding UGE findings in *HP*-associated gastritis, the Kyoto classification, which divides cases into three phases (uninfected, currently infected, and previously infected, including post-eradication), is currently used in clinical practice.^14^ In the Kyoto classification, gastric mucosal findings associated with active *HP* infection include atrophy, intestinal metaplasia, enlarged folds, nodularity, and diffuse redness; however, none of these were observed in the present case. In Japan, reports indicate that primary *HP* infection gastritis in adults frequently presents with upper abdominal symptoms and is associated with findings of acute gastric mucosal lesions (AGML).^15,16^ Regarding anti-*HP* antibodies, they are often negative during acute infection and are reported to become positive over time.^16^ This case was asymptomatic. UGE showed the presence of RAC, a finding indicative of uninfected gastric mucosa, and thin folds in the greater curvature of the gastric body, again suggesting uninfected *HP* status. Mild punctate erythema observed in the fundus and body regions may indicate early-stage gastric mucosal changes associated with *HP* infection.

At our health-screening facility, we perform a UBT on all patients prior to UGE. The accuracy of the UBT has been reported as 96.6% sensitivity and 96.9% specificity,^17^ and it was also found to be the most suitable for infection diagnosis in a meta-analysis of global *HP* guidelines.^18^ Moreover, Smart Gene, developed for detecting *HP* genes and clarithromycin resistance mutations, has an *HP* detection rate of 95.7%^19^ and is now widely used in Japan. Furthermore, regarding its diagnostic accuracy, compared with the UBT, stool antigen test, culture test, and real-time polymerase chain reaction, it has been reported to have a sensitivity of 100%, a specificity of 95.9%, and an overall agreement rate of 97.9%.^20^ During the UGE procedure, gastric fluid was collected, which revealed Smart Gene *HP* positivity and clarithromycin nonresistance, enabling a definitive diagnosis of active *HP* infection. Primary eradication of *HP* infection was successful, but no change was observed in the anti-*HP* antibody titers. The cutoff titer for diagnosing *HP* infection with this kit is ≥10 U/mL, but the recommended lower limit of sensitivity for this kit is 3 U/mL. The anti-*HP* antibody titer in this case was 5 U/mL. Titers within the range of 3 to 9.9 U/mL are classified as negative-high titer and have been reported to indicate a high-risk group for gastric cancer development.^21^ In negative-high titer cases with a high risk of gastric cancer development, advanced gastric mucosal atrophy is typically present; however, atrophic gastritis was not observed in the present case. Genetic differences between human hosts have been reported to potentially affect antibody levels in response to pathogens.^22^ Numerous genetic polymorphisms, including those for cytokines, metalloproteinases, glutathione transferases, and cyclooxygenase-2, are associated with host immune responses to *HP*.^23^ This case suggested an immune nonresponse to *HP*, as no change in antibody titers was observed before and after *HP* eradication, and no inflammatory changes were seen in the gastric mucosa. A previous report of initial *HP* infection in adults mostly involved abdominal symptoms and endoscopic findings consistent with AGML.^24^ The report confirmed persistent infection in 2 of 11 cases, while 9 cases were transient infections that were naturally cleared. The reason for the lack of elevated anti-*HP* antibody titers in this case of active *HP* infection is that, on the basis of the course of the disease to date and the UGE findings, the most likely scenario was that this case represented the initial stage of horizontal infection acquired in adulthood. At our clinic, the positive rate after introducing UBT was 12.4% (28/225), and this case was the only one in which no atrophic changes in the gastric mucosa were observed by UGE. The anti-*HP* antibody positivity rate among UBT-positive cases was 89.3% (25/28). Among these three cases, atrophic changes in the gastric mucosa on UGE were seen in the two cases other than our case. Very few health-screening facilities perform UBT on all examinees. This case was negative for anti-*HP* antibodies, and UGE revealed no findings suggestive of *HP* infection. Therefore, UBT and Smart Gene were considered highly useful for diagnosing active *HP* infection in this case. Although UGE is performed during general health checkups, the UBT is not conducted for *HP* infection, and the accumulation of additional cases is necessary.

In conclusion, a case of early-stage *HP* infection in adulthood without atrophic gastritis, diagnosed using the UBT and Smart Gene testing, was described.

## References

[1] Correa P, Piazuelo MB. The gastric precancerous cascade. J Dig Dis 2012; 13: 2–9. 22188910 10.1111/j.1751-2980.2011.00550.x PMC3404600

[2] Zamani M, Ebrahimtabar F, Zamani V, Miller WH, Alizadeh-Navaei R, Shokri-Shirvani J, Derakhshan MH. Systematic review with meta-analysis: the worldwide prevalence of Helicobacter pylori infection. Aliment Pharmacol Ther 2018; 47: 868–876. 29430669 10.1111/apt.14561

[3] Hooi JKY, Lai WY, Ng WK, Suen MMY, Underwood FE, Tanyingoh D, Malfertheiner P, Graham DY, Wong VWS, Wu JCY, Chan FKL, Sung JJY, Kaplan GG, Ng SC. Global prevalence of Helicobacter pylori infection: systematic review and meta-analysis. Gastroenterology 2017; 153: 420–429. 28456631 10.1053/j.gastro.2017.04.022

[4] Guo T, Zhou T, Zhu W, Yuan Y, Hui Y, Zhu W, Shen W, Li L, Wei W, Cheng H, Wu X. Burden and future trends of gastric cancer in 5 east Asian countries from 1990 to 2036: epidemiological study analysis using the Global Burden of Diseases Study 2021. JMIR Cancer 2025; 11: e74389. 40902202 10.2196/74389 PMC12408060

[5] Uemura N, Okamoto S, Yamamoto S, Matsumura N, Yamaguchi S, Yamakido M, Taniyama K, Sasaki N, Schlemper RJ. Helicobacter pylori infection and the development of gastric cancer. N Engl J Med 2001; 345: 784–789. 11556297 10.1056/NEJMoa001999

[6] Fukase K, Kato M, Kikuchi S, Inoue K, Uemura N, Okamoto S, Terao S, Amagai K, Hayashi S, Asaka M. Effect of eradication of Helicobacter pylori on incidence of metachronous gastric carcinoma after endoscopic resection of early gastric cancer: an open-label, randomised controlled trial. Lancet 2008; 372: 392–397. 18675689 10.1016/S0140-6736(08)61159-9

[7] Yokota S, Konno M, Fujiwara S, Toita N, Takahashi M, Yamamoto S, Ogasawara N, Shiraishi T. Intrafamilial, preferentially mother-to-child and intraspousal, Helicobacter pylori infection in Japan determined by mutilocus sequence typing and random amplified polymorphic DNA fingerprinting. Helicobacter 2015; 20: 334–342. 25664889 10.1111/hel.12217

[8] Take S, Mizuno M, Ishiki K, Imada T, Okuno T, Yoshida T, Yokota K, Oguma K, Kita M, Okada H, Yamamoto K. Reinfection rate of Helicobacter pylori after eradication treatment: a long-term prospective study in Japan. J Gastroenterol 2012; 47: 641–646. 22350696 10.1007/s00535-012-0536-9

[9] Katelaris P, Hunt R, Bazzoli F, Cohen H, Fock KM, Gemilyan M, Malfertheiner P, Mégraud F, Piscoya A, Quach D, Vakil N, Vaz Coelho LG, LeMair A, Melberg J. Helicobacter pylori World Gastroenterology Organization Global Guideline. J Clin Gastroenterol 2023; 57: 111–126. 36598803 10.1097/MCG.0000000000001719

[10] Fischbach W, Bornschein J, Hoffmann JC, Koletzko S, Link A, Macke L, Malfertheiner P, Schütte K, Selgrad DM, Suerbaum S, Schulz C. Update S2k-Guideline Helicobacter pylori and gastroduodenal ulcer disease of the German Society of Gastroenterology, Digestive and Metabolic Diseases (DGVS). Z Gastroenterol 2024; 62: 261–321. 38364851 10.1055/a-2181-2225

[11] Syam AF, Miftahussurur M, Makmun D, et al. Management of dyspepsia and Helicobacter pylori infection: the 2022 Indonesian Consensus Report. Gut Pathog 2023; 15: 25. 37217981 10.1186/s13099-023-00551-2 PMC10202071

[12] Smith SM, Boyle B, Buckley M, Costigan C, Doyle M, Farrell R, Ismail MS, Kevans D, Nugent S, O’Connor A, O’Morain C, Parihar V, Ryan C, McNamara D. The second Irish Helicobacter pylori Working Group consensus for the diagnosis and treatment of Helicobacter pylori infection in adult patients in Ireland. Eur J Gastroenterol Hepatol 2024; 36: 1000–1009. 38829956 10.1097/MEG.0000000000002796 PMC11198963

[13] Sun M, Liu E, Yang L, Cao H, Han M. A scoping review of worldwide guidelines for diagnosis and treatment of Helicobacter pylori infection. Syst Rev 2025; 14: 107. 40346683 10.1186/s13643-025-02816-0 PMC12063324

[14] Kamada T, Haruma K, Inoue K, Shiotani A. Helicobacter pylori infection and endoscopic gastritis -Kyoto classification of gastritis. Nihon Shokakibyo Gakkai Zasshi 2015; 112: 982–993 (in Japanese). 26050720 10.11405/nisshoshi.112.982

[15] Chatani N, Yada T, Ohkubob K, Aoki Y, Ogami T, Koizuka H, Uemura N, Ishida T. A case of acute gastric mucosal lesions secondary to primary infection with *H. pylori*. Progress of Digestive Endoscopy 2012; 81: 84–85 (in Japanese).

[16] Yagi K, Nakamura A, Sekine A. Two cases of AGML after initial Helicobacter Pylori infection, which was diagnosed by normal endoscopic finding, regular arrangement of collecting venules (RAC). Gastroenterological Endoscopy 2002; 44: 656–660 (in Japanese).

[17] Lemos FFB, de Castro CT, Silva Luz M, Rocha GR, Correa Santos GL, de Oliveira Silva LG, Calmon MS, Souza CL, Zarpelon-Schutz AC, Teixeira KN, Queiroz DMM, Freire de Melo F. Urea breath test for Helicobacter pylori infection in adult dyspeptic patients: A meta-analysis of diagnostic test accuracy. World J Gastroenterol 2024; 30: 579–598. 38463019 10.3748/wjg.v30.i6.579 PMC10921142

[18] Sun M, Liu E, Yang L, Cao H, Han M. A scoping review of worldwide guidelines for diagnosis and treatment of Helicobacter pylori infection. Syst Rev 2025; 14: 107. 40346683 10.1186/s13643-025-02816-0 PMC12063324

[19] Kakiuchi T, Okuda M, Matsuo M, Fujimoto K. Smart Gene™ as an effective non-invasive point-of-care test to detect Helicobacter pylori clarithromycin-resistant mutation. J Gastroenterol Hepatol 2022; 37: 1719–1725. 35562319 10.1111/jgh.15887

[20] Tsuda M, Watanabe Y, Oikawa R, Watanabe R, Higashino M, Kubo K, Yamamoto H, Itoh F, Kato M. Clinical evaluation of a novel molecular diagnosis kit for detecting Helicobacter pylori and clarithromycin-resistant using intragastric fluid. Helicobacter 2022; 27: e12933. 36263754 10.1111/hel.12933 PMC9788249

[21] Kishikawa H, Kimura K, Takarabe S, Kaida S, Nishida J. Helicobacter pylori antibody titer and gastric cancer screening. Dis Markers 2015; 2015: 156719. 26494936 10.1155/2015/156719 PMC4606161

[22] Rubicz R, Leach CT, Kraig E, Dhurandhar NV, Duggirala R, Blangero J, Yolken R, Göring HH. Genetic factors influence serological measures of common infections. Hum Hered 2011; 72: 133–141. 21996708 10.1159/000331220 PMC3214928

[23] Toyoshima O, Nishizawa T, Arita M, Kataoka Y, Sakitani K, Yoshida S, Yamashita H, Hata K, Watanabe H, Suzuki H. Helicobacter pylori infection in subjects negative for high titer serum antibody. World J Gastroenterol 2018; 24: 1419–1428. 29632423 10.3748/wjg.v24.i13.1419 PMC5889822

[24] Suehiro M, Haruma K, Kamada T, Oka T, Ishii K, Katsumata R, Tanikawa T, Urata N, Sasai T, Fujita M, Ayaki M, Manabe N, Kawamoto H, Monobe Y. Two Cases of Acute Gastric Mucosal Lesions Due to Helicobacter pylori Infection Confirmed to be Transient Infection. Intern Med 2023; 62: 381–386. 35676034 10.2169/internalmedicine.8741-21 PMC9970821

